# New Anti-Spasmodic—Sumbul

**Published:** 1850-07

**Authors:** 


### MATERIA MEDICA AND THERAPEUTICS.

*New Anti-spasmodic—Sumbul.*—In recently attending the medical wards of King's College Hospital, we perceived that Dr. Todd was prescribing, in a case of epilepsy, a medicine, the name of which we heard for the first time. On inquiry, we find that this root, called *sumbul*, is being introduced into practice as an anti-spasmodic by Mr. Savory of Bond street. It appears that Dr. Granville, on a recent return from the continent, mentioned sumbul to Mr. Savory as a root employed with great success in Germany and Russia against cholera: wondering that it had not yet found its way into this country. Mr. Savory immediately set about procuring it, but his correspondent in Russia had much trouble in getting this root; he however sent over specimens of it. A little later, another parcel of the sumbul was obtained by the same house from Hamburg; and on comparing the two samples, Mr. Savory came to the conclusion that the German one was decidedly the firmest, least damaged, and in the best condition of the two. We were shown these specimens, and find that they much resemble the circular pieces in which calumba is generally seen, except that they are considerably larger, of a more spongy texture, and resembling huge bungs. They are of a yellowish gray, whitish in the centre, with a thin, pellicular bark surrounding them. The most striking feature of the root is its very strong odor, which very much approaches that of musk, being almost as pleasant and powerful. The pieces are very light, and seemed to be formed of a condensed and hardened pithy substance. From this imperfect description, it may at once be gathered that the sumbul promised to be useful as an anti-spasmodic, and Mr. Savory first thought of combining it with the cotyledon umbilicus, and using it against epilepsy. It is, however, being tried by itself, and though Dr. Todd cannot as yet state anything positive as to results, we were told that the little boy who is taking ten minims of the tincture thrice a day, and who, when admitted, had an epileptic fit once or twice a week, has had no attack since he has been in the hospital. Mr. Savory has likewise prepared an extract of the root, and further trials are of course necessary to judge of the efficacy of the medicine. Musk being, however, very expensive, it would be a great boon to the public were this root found as efficacious. Nothing is yet known of the botanical origin of the plant; efforts are, however, being made to ascertain its natural history, and we shall have much pleasure in communicating to our readers the details which may transpire, both in the latter respect and with regard to the trials which are being made as to the therapeutical virtues of the root.—*Dublin Med. Press, from the Lancet.*

